# An examination of Alzheimer’s disease and white matter from 1981 to 2023: a Bibliometric and visual analysis

**DOI:** 10.3389/fneur.2023.1268566

**Published:** 2023-11-14

**Authors:** Linman Wu, Liuyin Jin, Lixia Li, Kai Yu, Junnan Wu, Yuying Lei, Shulan Jiang, Jue He

**Affiliations:** ^1^School of Mental Health, Wenzhou Medical University, Wenzhou, China; ^2^Nanchong Mental Health Center of Sichuan Province, Nanchong, China; ^3^Lishui Second People’s Hospital, Wenzhou Medical University, Lishui, China

**Keywords:** Alzheimer’s disease, white matter, Bibliometrics, VOSviewer, CiteSpace

## Abstract

**Background:**

Alzheimer’s disease (AD) is characterized by the presence of gray matter lesions and alterations in white matter. This study aims to investigate the research related to white matter in the context of AD from a Bibliometric standpoint.

**Methods:**

Regular and review articles focusing on the research pertaining to Alzheimer’s disease (AD) and white matter were extracted from the Web of Science Core Collection (WOSCC) database, covering the period from its inception to 10th July 2023. The “Bibliometrix” R package was employed to summarize key findings, to quantify the occurrence of top keywords, and to visualize the collaborative network among countries. Furthermore, VOSviewer software was utilized to conduct co-authorship and co-occurrence analyses. CiteSpace was employed to identify the most influential references and keywords based on their citation bursts. The retrieval of AD- and white matter-related publications was conducted by the Web of Science Core Collection. Bibliometric analysis and visualization, including the examination of annual publication distribution, prominent countries, active institutions and authors, core journals, co-cited references, and keywords, were carried out by using VOSviewer, CiteSpace, the Bibliometrix Package, and the ggplot2 Package. The quality and impact of publications were assessed using the total global citation score and total local citation score.

**Results:**

A total of 5,714 publications addressing the intersection of Alzheimer’s disease (AD) and white matter were included in the analysis. The majority of publications originated from the United States, China, and the United Kingdom. Prominent journals were heavily featured in the publication output. In addition to “Alzheimer’s disease” and “white matter,” “mild cognitive impairment,” “MRI” and “atrophy” had been frequently utilized as “keywords.”

**Conclusion:**

This Bibliometric investigation delineated a foundational knowledge framework that encompasses countries, institutions, authors, journals, and articles within the AD and white matter research domain spanning from 1981 to 2023. The outcomes provide a comprehensive perspective on the broader landscape of this research field.

## 1 Introduction

Alzheimer’s disease (AD), a prevailing form of dementia, is a progressive neurodegenerative disorder initially characterized and named after Alois Alzheimer, a German teacher, in 1906. It is characterized by a gradual decline in memory and other cognitive functions. The primary pathological features of AD encompass neurofibrillary tangles (NFTs), senile plaques (SP), and significant neuronal loss. Amyloid precursor protein (APP) undergoes degradation by α-, β-, and γ-proteases, with the latter two resulting in the breakdown of APP and the generation of β-amyloid protein (Aβ). The toxic form of Aβ leading to neural cell apoptosis plays a critical role in promoting SP formation in the brain, and is widely acknowledged as a significant contributor to the pathogenesis of AD (1).

Currently, the etiology of AD remains incompletely understood, and it is conventionally regarded as a disorder primarily affecting gray matter. However, in recent years, numerous neuroimaging investigations have revealed that apart from neuronal structural impairment, white matter degeneration and demyelination may also constitute significant pathophysiological characteristics (2, 3). White matter comprises neuronal axons and myelin sheaths enveloping the axons. Myelin sheaths generated by oligodendrocytes (OLs) play a crucial role in maintaining the structural integrity of brain and proper functioning of neurons. Due to the crucial function of the oligodendrocyte cell lineage in both myelin production and the process of remyelination, alterations in the quantity of oligodendrocytes or their precursor cells, as well as any impairment in their function, may have an impact on the integrity of myelin. Mounting evidence suggests that alterations in oligodendrocytes occur during the pathogenesis of AD (4–8). Therefore, white matter, myelination and oligodendrocytes play an important role in the development of AD.

According to a 2018 estimation by Alzheimer’s Disease International, the global prevalence of dementia was approximately 50 million individuals, with projections indicating a threefold increase by 2050. It is noteworthy that two-thirds of these cases are concentrated in low- and middle-income nations (9). AD imposes a significant economic burden on families and society, and the absence of an effective treatment underscores the continued research focus on unraveling the pathogenesis of AD white matter and identifying efficacious therapeutic interventions.

Bibliometrics, a subfield of informatics, involves the quantitative and qualitative analysis of literature, focusing on the characteristics and system of literature itself. This methodology enables the quantitative assessment of the distribution, interrelationships, and clustering within a specific research field (10). Furthermore, Bibliometrics has emerged as a prominent methodology for evaluating the authenticity, excellence, and influence of scholarly endeavors (11, 12). Bibliometrics allows for the evaluation of the contributions and influence of diverse authors, countries, institutions, disciplines, and journals. Moreover, it facilitates the examination of the status, patterns, and forefront of research endeavors (13). In order to discover valuable insights and references for investigating the pathogenesis of white matter in Alzheimer’s disease, this study conducted a comprehensive quantitative and visual analysis of the pertinent global literature on white matter research in Alzheimer’s disease from 1981 to 2023.

## 2 Methods

### 2.1 Data acquisition and search strategy

**Figure 1:**
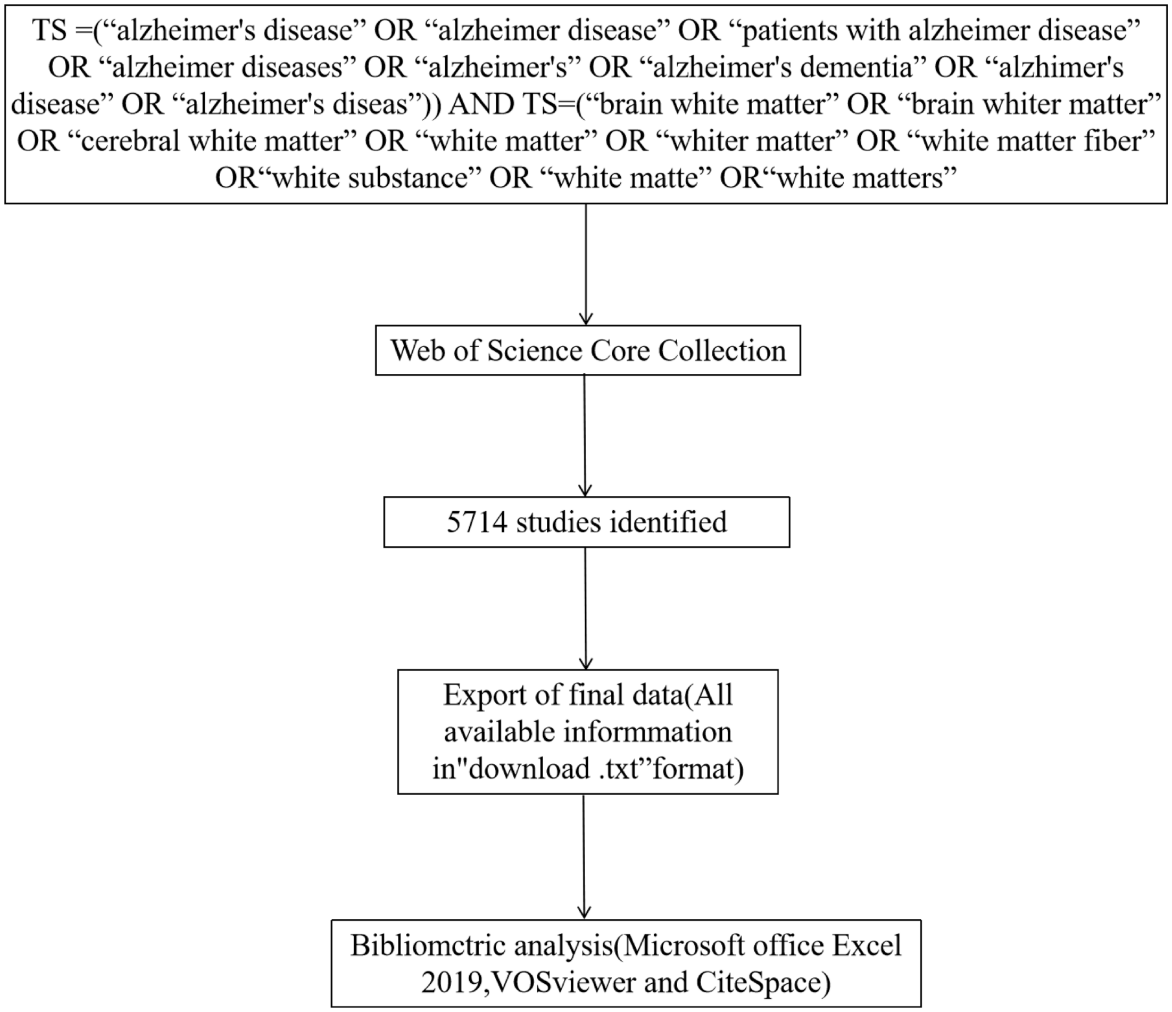
The flowchart illustrating the search strategy and selection process in AD and white matter.

Web of Science (WOS) is one of the most commonly used sources of scholarly databases, containing more than 12,000 influential journals and an independent global citation database from the world’s most trusted publishers (14). To improve data representativeness and accessibility, we retrieved the Web of Science Core Collection (WoSCC) database. As shown in Figure 1, the search query employed was as follows: Topic search (TS) = (“alzheimer’s disease” OR “alzheimer disease” OR “patients with alzheimer disease” OR “alzheimer diseases” OR “alzheimer’s” OR “alzheimer’s dementia” OR “alzhimer’s disease” OR “alzheimer’s diseas”) AND TS = (“brain white matter” OR “brain whiter matter” OR “cerebral white matter” OR “white matter” OR “whiter matter” OR “white matter fiber” OR “white substance” OR “white matte” OR “white matters”). The search was restricted to English language articles and document types such as Review Articles and Articles. The document information was saved in TXT format. To ensure data accuracy and avoid any inconsistencies resulting from updates, all the aforementioned procedures were conducted within a single day, specifically on 10th July 2023.

### 2.2 Eligibility criteria and data collection

Only articles and reviews were deemed eligibly for inclusion in this study. Materials such as meeting abstracts, editorial pieces, and proceedings papers were excluded from the analysis. To ensure accuracy, any duplicate studies were identified and removed manually. The collected data encompassed various Bibliometric elements, including the number of papers and citations, titles, authors, affiliations, countries, keywords, journals, publication years, and references.

### 2.3 Statistical analysis

For the Bibliometric analysis in this study, we utilized the Bibliometrix 4.1.0 Package^1^ and the ggplot2 Package 3.3.6, implemented in the R programming language. To visualize complex co-citation networks, VOSviewer 1.6.19 was employed. In the visualizations, the size of the nodes indicates the number of publications, the line thickness represents the strength of the connection, and the node colors denote different clusters or time periods (15).

CiteSpace software was employed to facilitate the visual analysis of the knowledge domain and emerging trends (16). This included cluster analysis, dual-map overlay of citations, timeline or time zone views, examination of references, and identification of citation bursts for keywords (17, 18). Cluster analysis plays a crucial role in classifying references and keywords, facilitating the identification of significant research areas in the field of AD. Two vital evaluation metrics in cluster analysis are modularity Q and mean silhouette. A value of Q > 0.3 suggests a substantial clustering structure, while a mean silhouette value > 0.5 indicates reliable clustering outcomes. Keyword and reference bursts are frequently utilized to uncover emerging research trends within the field.

The Bibliometrix Package, a well-established tool based on the R programming language, is employed for conducting Bibliometric analysis (19). In this study, the Bibliometrix Package was used to perform a thematic evolution analysis, enabling the categorization of podocyte injury research into distinct periods.

## 3 Results

### 3.1 Analysis of annual publications

Following the exclusion of non-English articles and non-review or monograph types, a total of 5,714 articles from 1981 to 2023 were identified in the Web of Science database. Among them, 4,588 articles fell under the category of literature, while 889 articles were classified as reviews.

**Figure 2:**
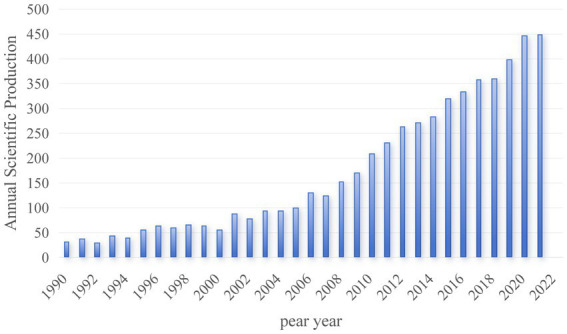
Temporal distribution map of the literature. The blue bars in the graph represent the annual publication count, with each bar corresponding to the number of publications in a specific year.

Figure 2 delineates the trajectory of annual publication counts and trends in Alzheimer’s Disease (AD) and white matter research spanning from 1990 to 2022. Over this period, there has been a substantial surge in the scholarly output within this domain, progressing from a mere three publications annually in the 1990s to an impressive tally of 449 *per annum* in 2022, underscoring a marked and rapid growth trend.

### 3.2 Analysis of countries and international collaboration

**Figure 3:**
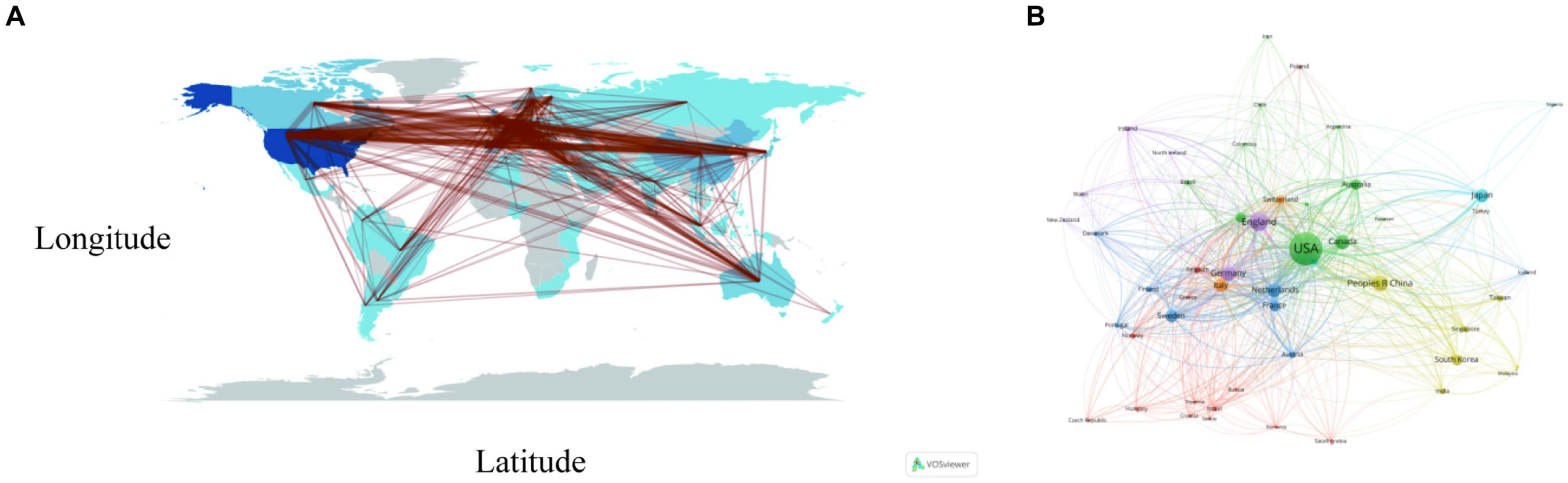
Visualization of countries that performed research on AD and white matter. **(A)** The collaboration between countries and regions on nutrition research related to sarcopenia. The color segmentation includes blue (with publications) and gray (without publications). The thickness of the red lines indicates the number of co-published papers. The color intensity corresponds to the number of publications. **(B)** Where SCP represents bilateral cooperation and MCP represents multilateral cooperation, the height of the bar chart represents the number of collaborations, with the color changing from blue to red indicating an increase in the number of collaborations.

Figure 3A portrays the dissemination of scholarly articles across nations and regions. A deeper hue signifies a higher count, with the United States leading in publications (*N* = 1,850, 32.38%), succeeded by China (*N* = 499, 8.73%), the United Kingdom (*N* = 433, 7.58%), and Japan (*N* = 318, 5.57%). All other nations accumulated fewer than 300 articles (Figure 3A; Table 1). Both Figures 3A,B elucidate the realms of international collaboration among nations. Such collaborations markedly catalyze the advancement of scientific exploration. Predominantly, research partnerships manifest amidst nations of North America, Europe, Oceania, and East Asia. Remarkably, the United States and the United Kingdom engage in the most frequent collaborations (frequency = 227), trailed by collaborations between the United States and China (frequency = 174), and the United States and Germany (frequency = 140). The lines in the figures symbolize collaboration: a higher count of lines indicates a more robust collaboration (Figures 3A,B; Table 2). However, the majority of nations struggle to forge a path, signifying a dearth of consistent interchanges and collaborative endeavors.

**Table 1:** The number of published articles from the top 10 countries.

Rank	Country	Articles	% of (5,714)	Citations
1	USA	1,850	32.38	156,412
2	China	499	8.73	14,212
3	United Kingdom	433	7.58	48,558
4	Japan	318	5.57	13,674
5	Canada	279	4.88	20,728
6	Netherlands	252	4.41	27,558
7	Italy	235	4.11	17,258
8	Germany	231	4.04	23,966
9	Korea	227	3.97	6,424
10	Sweden	176	3.08	21,812

**Table 2:** The frequency of collaboration among the top 10 ranked countries.

From	To	Frequency
USA	United Kingdom	227
USA	China	174
USA	Germany	140
USA	Canada	138
United kingdom	Sweden	125
United kingdom	Netherlands	124
United kingdom	Germany	113
USA	Netherlands	108
USA	Sweden	101
United Kingdom	Italy	86

### 3.3 Analysis of institutions

**Figure 4:**
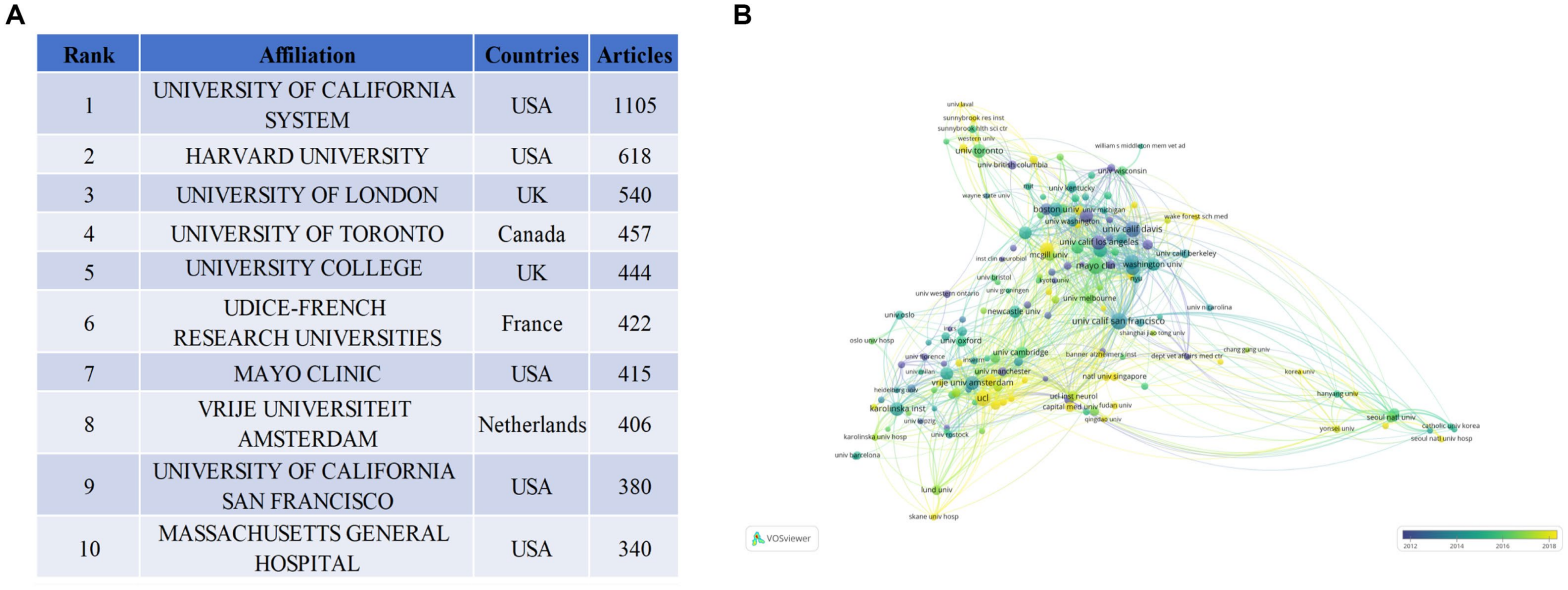
Visualization of institutions that performed research on AD and white matter. **(A)** Total publications of the top 10 ranked institutions. **(B)** Institution collaboration network graph, with a minimum publication threshold of 20 articles; 162 institutions and authors were selected.

Approximately 4,783 entities have engaged in research related to Alzheimer’s Disease (AD) and white matter. Figure 4A presents a roster of the top 10 most productive institutions, with the United States boasting five of the highest producers, followed by two from the United Kingdom. The UNIVERSITY OF CALIFORNIA SYSTEM leads with a publication count of 1,105, securing the top position, followed by HARVARD UNIVERSITY (*N* = 618), UNIVERSITY OF LONDON (*N* = 540), UNIVERSITY OF TORONTO (*N* = 457), and UNIVERSITY COLLEGE LONDON (*N* = 444). Collaborative analyses based on affiliations can estimate the relationships between different institutions by assessing the quantity of co-published works. In the coverage network of co-authorship analysis depicted in Figure 4B, the size of the circles represents the publication volume, and the color signifies the average publication commencement year within the specific research domain for each institution. As demonstrated in Figure 4B, a total of 162 institutions have been identified, each having published a minimum of 20 articles. Researchers from HARVARD UNIVERSITY embarked on AD and white matter research relatively early. Conversely, investigators from Harvard Medical School have delved into this domain more recently. The data illustrates close collaboration among these institutions.

### 3.4 Analysis of authors

**Figure 5:**
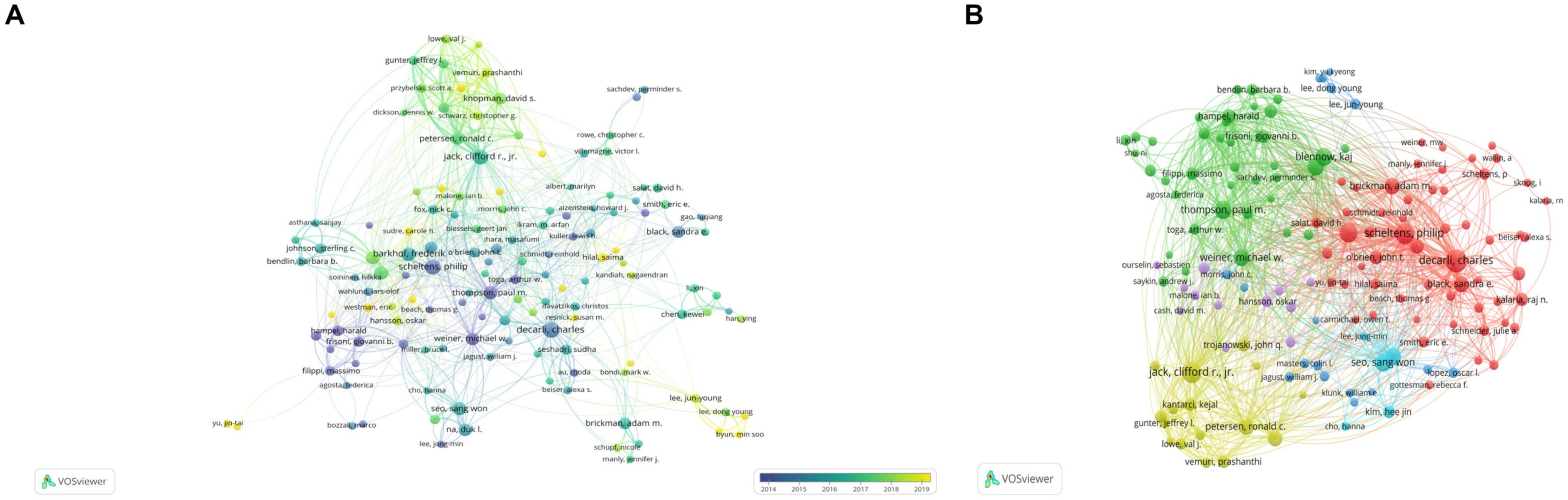
Illustrates authors associated with research on Alzheimer’s Disease (AD) and white matter. **(A)** Depicts co-occurrence of authors. **(B)** Presents a VOSviewer visualization of co-cited authors. The size of the circles corresponds to the quantity of articles authored by each individual, while the interconnecting lines symbolize their collaborative exchanges and interactions.

As shown in Table 3, a total of 15,692 authors have contributed to publications related to Alzheimer’s Disease (AD) and white matter. The team/lab led by SCHELTENS P exhibits the highest influence, boasting an h-index of 50 and a publication count of 107 articles. Following closely are DECARLI C (*N* = 105, h-index = 49), KALARIA RN (*N* = 62, h-index = 43), and JACK CR (*N* = 47, h-index = 28). Figure 5 represents a collaborative network among researchers, with a minimum paper count set at 15 for each author. Among the remaining 144 authors, several communities have emerged, each gravitating around a few prolific authors who publish frequently. The connections between these distinct communities appear relatively sparse, indicating that robust collaborative relationships among research teams/laboratories engaged in AD- and white matter-related studies are yet to be fully established.

**Table 3:** The publication volume and impact factor of the top 10 ranked authors.

Rank	Author	h_index	TC	NP	PY_start
1	SCHELTENS P	50	8,502	107	1992
2	DECARLI C	49	10,950	105	1996
3	KALARIA RN	43	7,529	62	1992
4	JACK CR	40	6,890	102	1998
5	WEINER MW	39	6,339	79	1994
6	BARKHOF F	36	4,648	87	1992
7	BLENNOW K	34	4,677	74	1991
8	TROJANOWSKI JQ	34	5,583	47	1992
9	THOMPSON PM	33	4,953	58	1998
10	HAMPEL H	32	3,302	53	1998

### 3.5 Analysis of journals

A total of 918 journals have published articles on Alzheimer’s Disease (AD) and white matter research. Table 4 displays the top 10 journals with the highest publication counts and their recent Impact Factors (IF). Given their extensive coverage of relevant topics, these journals are more likely to accept articles related to AD and white matter research. It is recommended that scholars focusing on AD and white matter research pay particular attention to these journals. JOURNAL OF ALZHEIMERS DISEASE leads with the highest publication count (*N* = 445, IF = 4.0), followed by NEUROBIOLOGY OF AGING (*N* = 245, IF = 4.2), NEUROLOGY (*N* = 180, IF = 9.9), FRONTIERS IN AGING NEUROSC IENCE (*N* = 166, IF = 4.8), and NEUROIMAGE (N = 128, IF = 5.7). Notably, 40% of the journals fall into Q1, and 50% fall into Q2, with 60% categorized under Neurology, indicating the generally high quality of research. In terms of publication origin, among the top 10 journals, 4 are from the United States, 3 from the Netherlands, 2 from Switzerland, and 1 from the United Kingdom.

**Table 4:** The top 10 journals by publication volume and impact factor.

Rank	Sources	Articles	Country	IF	JCR-c
1	JOURNAL OF ALZHEIMERS DISEASE	445	Netherlands	4.0	Q2
2	NEUROBIOLOGY OF AGING	245	England	4.2	Q2
3	NEUROLOGY	180	United States	9.9	Q1
4	FRONTIERS IN AGING NEUROSCIENCE	166	Switzerland	4.8	Q1
5	NEUROIMAGE	128	United States	5.7	Q1
6	ALZHEIMERS & DEMENTIA	107	United States	14.0	Q1
7	NEUROIMAGE-CLINICAL	97	Netherlands	4.2	Q2
8	JOURNAL OF THE NEUROLOGICAL SCIENCES	95	Netherlands	4.4	Q2
9	DEMENTIA AND GERIATRIC COGNITIVE DISORDERS	88	Switzerland	2.4	Q3
10	PLOS ONE	87	United States	3.7	Q2

IF, impact factor (2022–2023); JCR-c, Journal Citation Reports category (2023).

Through citation analysis, the identification of the most frequently cited articles provides valuable insights into the current research hotspots within the respective field. Utilizing Bibliometrix, we obtained the top 10 highly cited articles (Table 5). Among them, the article titled “Brain infarction and the clinical expression of Alzheimer’s disease. The Nun Study” by Snowdon DA, published in JAMA-J AM MED ASSOC in 1997, exhibited the highest citation frequency, with 337 local citations and 1,878 global citations. The second-ranked article was authored by Bozzali M in 2002, titled “White matter damage in Alzheimer’s disease assessed *in vivo* using diffusion tensor,” published in J NEUROL NEUROSUR PS, with 236 local citations and 435 global citations. Ranking third, Gorelick PB’s article “Vascular contributions to cognitive impairment and dementia: a statement for healthcare professionals from the American Heart Association/American Stroke Association,” published in J STROKE in 2011, amassed 224 local citations and an impressive 2,339 global citations. The remaining articles are listed in Table 5.

**Table 5:** Top 10 cited articles for in the field of AD and white matter research.

Number	First author	Article name	Journal name	Year	Local citations	Global citations	LC/GC ratio (%)
1	Snowdon DA	Brain infarction and the clinical expression of Alzheimer’s disease. The Nun Study	JAMA-J AM MED ASSOC	1997	337	1,878	17.94
2	Bozzali M	White matter damage in Alzheimer’s disease assessed *in vivo* using diffusion tensor magnetic resonance imaging	J NEUROL NEUROSUR PS	2002	236	435	54.25
3	Gorelick PB	Vascular contributions to cognitive impairment and dementia: a statement for healthcare professionals from the American heart association/American stroke association	STROKE	2011	224	2,339	9.58
4	Zhang Y	Diffusion tensor imaging of cingulum fibers in mild cognitive impairment and Alzheimer’s disease	NEUROLOGY	2007	220	417	52.76
5	Medina D	White matter changes in mild cognitive impairment and AD: a diffusion tensor imaging study	NEUROBIOL AGING	2006	217	385	56.36
6	Klunk WE	Imaging brain amyloid in Alzheimer’s disease with Pittsburgh Compound-B	ANN NEUROL	2004	215	3,226	6.66
7	Bartzokis G	Age-related myelin breakdown: a developmental model of cognitive decline and Alzheimer’s disease	NEUROBIOL AGING-a	2004	187	715	26.15
8	Lee S	White matter hyperintensities are a core feature of Alzheimer’s disease: Evidence from the dominantly inherited Alzheimer network	ANN NEUROL	2016	177	294	60.20
9	Rose SE	Loss of connectivity in Alzheimer’s disease: an evaluation of white matter tract integrity with color coded MR diffusion tensor imaging	J NEUROL NEUROSUR PS	2000	169	376	44.95
10	Prins ND	White matter hyperintensities, cognitive impairment and dementia: an update	NAT REV NEUROL	2015	162	614	26.38

### 3.6 Analysis of keywords

**Figure 6:**
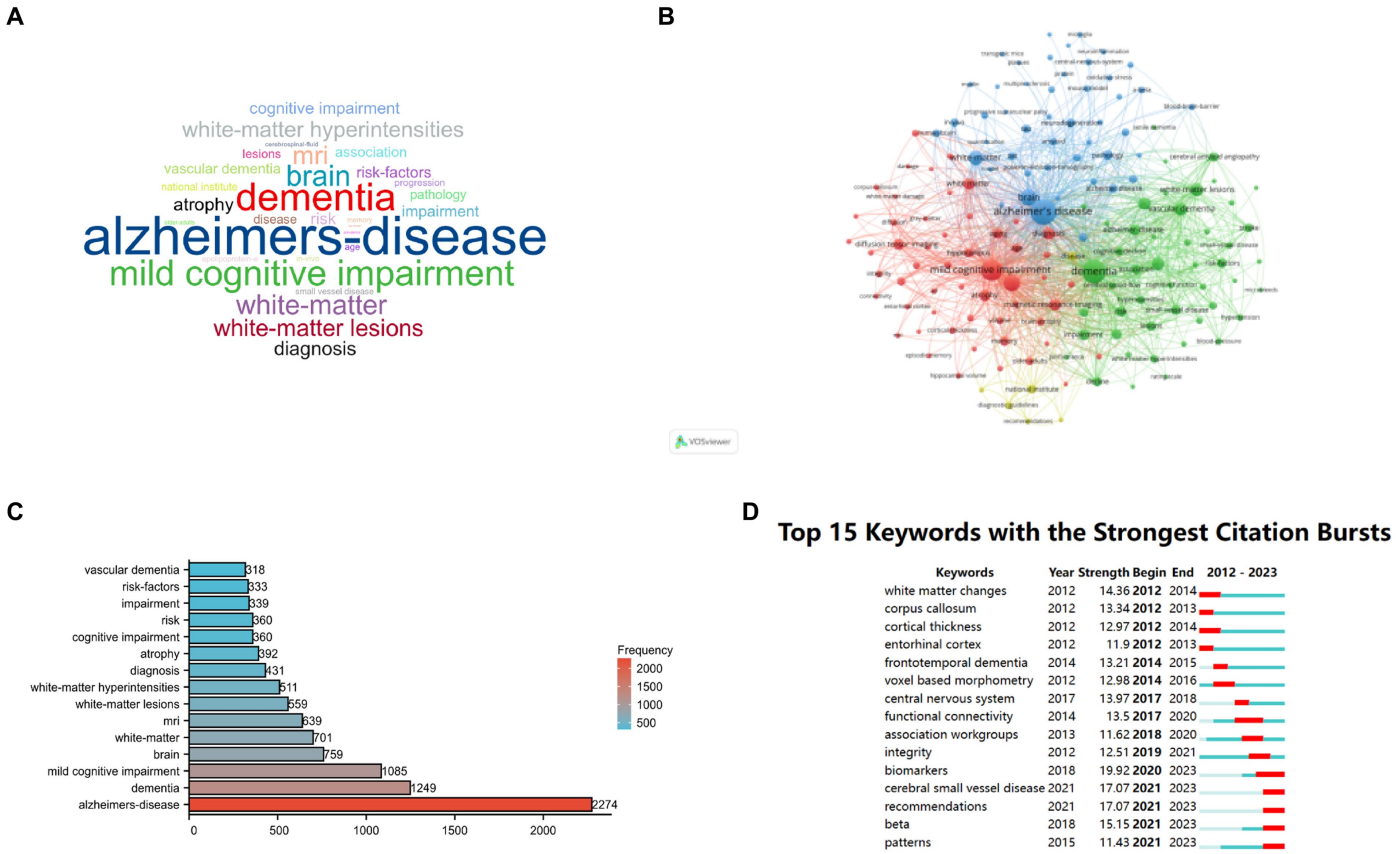
Showcases an in-depth analysis of research keywords related to Alzheimer’s Disease (AD) and white matter. **(A)** Presents a network visualization of author-generated keywords; **(B)** Offers an overlay visualization of these keywords; **(C)** Highlights the top 10 most frequently occurring keywords; **(D)** Provides a visualization map featuring the top 20 keywords exhibiting the most substantial bursts of citations.

To visually illustrate the prominent keywords in the field of medical image segmentation, word clouds were generated based on the extracted author keywords. Figure 6A exhibits the word cloud, where the font size corresponds to the frequency of each word or phrase.

Utilizing VOSviewer software, a keyword co-occurrence analysis was conducted, specifically employing “Keywords Plus” and setting the connection degree threshold to 70. As a result, 155 keywords were identified, forming four distinct clusters represented by colors: red, green, blue, and yellow (Figure 6B).

From the comprehensive pool of 7,812 keywords obtained through Bibliometrix, the top 10 most recurrent ones were identified, led by “Alzheimer’s disease” with a frequency of 2,274, followed by “dementia” (frequency = 1,249), “mild cognitive impairment” (frequency = 1,085), “brain” (frequency = 759), and “white-matter” (frequency = 701). Other notable terms include “MRI” (frequency = 639), “white-matter lesions” (frequency = 559), “white-matter hyperintensities” (frequency = 511), “diagnosis” (frequency = 431), and “atrophy” (frequency = 492). The top 30 keywords are visualized in the tree view (Figure 6C).

Furthermore, Figure 6D shows the top 15 keywords with the most robust citation bursts observed during the period from 2012 to 2023. Notably, substantial attention have been gained on keywords including “biomarkers” (2020–2023), “cerebral small vessel disease” (2021–2023) and “recommendations” (2021–2023) in recent years, indicating their potential as emerging and pivotal research topics in the future.

## 4 Discussion

In this investigation, we employed advanced **B**ibliometric methodologies to scrutinize the evolutionary trajectories of research concerning Alzheimer’s disease (AD) and cerebral white matter spanning from 1981 to 2023. Notably, the corpus of publications pertaining to AD and cerebral white matter exhibited a persistent upward surge after 2009. Analogously, a previous Bibliometric inquiry exclusively dedicated to AD and white matter in magnetic resonance imaging (MRI) revealed a comparable pattern, indicating a pronounced escalation in MRI-focused investigations related to AD (20).

This upward trajectory of scholarly endeavors is plausibly predicated on the burgeoning recognition of the intimate interplay between AD etiology and cerebral white matter pathogenesis. The alterations in cerebral white matter volume as shown by MRI signal patterns could tie inextricably to the emergence and progression of AD (21, 22). The analysis further revealed that the preponderance of publications pertaining to AD and cerebral white matter emanated from authors affiliated with diverse nations, including the United States, China, the United Kingdom, and Canada. Concomitantly, analogous trends have been discerned across other fields of Bibliometric research, spanning schizophrenia (23), autism (24), and Parkinson’s disease (25). Such academic development intimately correlates with a nation’s economic prowess (26), thereby accentuating that advancements in medical research are closely intertwined with governmental support in the healthcare domain. Evidently, the United States’ robust healthcare investments surpassing those of other nations potentially elucidate the concentration of AD and cerebral white matter research publications in this region (27, 28). Moreover, it is notable that most collaborative efforts concerning AD and cerebral white matter are anchored in the United States, seamlessly aligning with its pivotal contributions to this domain of research, while collaborative endeavors between other nations necessitate further strengthening.

Delving into the publication landscape across various journals, it emerged that 445 articles pertaining to AD and cerebral white matter found their scholarly abode in the esteemed Journal of Alzheimer’s Disease. Journal of Alzheimer’s Disease, an academic venue whose genesis dates back to 1998, is edited in the Netherlands and circulated in Washington. At present, the journal boasts an impressive impact factor of 4. Distinguished by its thematic focus on AD pathogenesis and therapeutic interventions, the Journal of Alzheimer’s Disease has recently witnessed noteworthy investigations delving into the ramifications of compounds such as resveratrol, kaempferol, and Eduesmin on AD pathophysiology (29–32). In essence, the number of articles published by research collectives serves as a testament to their active engagement and contributions within a given scientific sphere. Notably, the University of California system emerged as the most prolific contributor to AD and cerebral white matter research, accentuating the indispensable role played by the United States in propelling this field of inquiry (28, 33).

Efficiently deciphering research hotspots entails discerning the prevailing thematic focal points in a given domain during a defined period. Acquaintance with research hotspots proves advantageous for researchers as it allows them to be well-attuned to the contemporaneous trends within their specific fields of interest, thus constituting one of the prime objectives underlying Bibliometric analyses (34). In the case of AD and cerebral white matter research, our investigation pinpointed the primary research thrusts, prominently encompassing the elucidation of the intricate interconnections between AD and cerebral white matter, investigations into cerebral white matter’s MRI signal alterations, and inquiries delving into cognitive impairment and diagnostic aspects associated with this formidable neurological disorder (35–37).

Citation analysis can reflect the academic influence of publications. Among the 10 most cited articles in this study, the focus remains centered on research exploring the relationship between Alzheimer’s disease (AD) and white matter signal alterations and cognitive impairment. Notably, recent research indicates that the incidence of AD gradually increases with age, but underdiagnosis and misdiagnosis are prevalent. The risk of dementia escalates as the disease progresses, but the transition from normal cognition and absence of Aβ accumulation to proteinopathy and cognitive impairment takes time. Consequently, early biological markers of cognitive impairment in AD patients play a crucial role in diagnosis. A plethora of studies has found close associations among AD, cognitive impairment, the tau/Aβ ratio, APOE4 positive status, white matter hyperintensities, and atrophy in specific brain regions, and these may serve as potential diagnostic biomarkers of AD in the future (22, 35–40).

Keywords in scientific research signify The core subjects within a research domain. Bibliometric analysis of Dendritic Epidermal T Cell research from 1983 to 2019 is an essential metric in scientific investigation. Co-occurrence analysis of keywords reveals the interconnectedness and popularity of research topics within the scientific field. Among the most frequently appearing keywords, apart from “Alzheimer’s” and “white matter,” other commonly used keywords predominantly focus on aspects related to cognitive impairment and white matter signal alterations.

The co-occurrence clustering function divides the entire network into approximately four clusters each representing a distinct theme. Cluster 1 denoted in green encompasses research concerning changes in brain white matter volume and hippocampal alterations. Studies have shown that AD is associated with reduced volumes in the left parahippocampal gyrus right temporal white matter extending to the parahippocampal gyrus and the posterior corpus callosum. Affecting memory formation white matter atrophy particularly in regions adjacent to bilateral structures such as hippocampus amygdala and entorhinal cortex is a common feature in AD (41–43).

Cluster 2, depicted in red, includes several terms related to AD diagnostic biomarkers, such as Aβ, microglia, and neuroinflammation. Research has revealed activated microglia and elevated levels of inflammatory markers in AD patients, along with changes in risk genes associated with innate immunity. Neuroinflammation plays a critical role in the pathogenesis of AD, making the study of its association with AD a prominent research hotspot, potentially relevant to the diagnosis and treatment of AD (44–46).

Surge detection analysis is an essential approach to explore the evolution of research hotspots within academic domains. High-cited articles or keywords signify active discussions or usage during specific periods. Since 2020, AD diagnostic biomarkers and Aβ have consistently emerged as surge keywords, indicating their prominence in the research landscape. Currently, the investigation of AD biomarker Aβ as a potential therapeutic target remains a major research focus (47, 48).

Several limitations are associated with this study. Firstly, the data were retrieved solely from WOSCC. Although WOS is recommended as a reliable database for Bibliometric research, this limitation should be considered in interpreting the findings (49–52).

However, it is important to acknowledge several limitations in this study. Firstly, it is possible that some articles may have been inadvertently omitted despite our efforts to be comprehensive. Secondly, our restriction to English-language literature could introduce a language bias in the selection of publications. Thirdly, over time, institutions and journal names may undergo changes, leading to potential biases in our literature search.

## 5 Conclusion

In conclusion, our research illuminates the dynamic landscape of AD and brain white matter research, and shows the interdisciplinary nature of the field. By highlighting the significance of biomarkers and white matter alterations in AD, our findings provide valuable insights that can inform future investigations and therapeutic strategies. We hope that this study will inspire further collaboration and exploration, ultimately advancing our understanding of AD and driving progress toward effective interventions and treatments.

## Data availability statement

The raw data supporting the conclusions of this article will be made available by the authors, without undue reservation.

## Author contributions

LW: Methodology, Writing – original draft. LJ: Formal analysis, Methodology, Writing – original draft. LL: Data curation, Writing – review & editing. KY: Data curation, Writing – review & editing. JW: Data curation, Writing – review & editing. YL: Validation, Writing – review & editing. SJ: Supervision, Writing – review & editing. JH: Funding acquisition, Supervision, Writing – review & editing.

## Footnotes

^1^ https://www.bibliometrix.org
